# Platelet to Lymphocyte Ratio and Neutrophil to Lymphocyte Ratio in Missed Abortion

**DOI:** 10.1055/s-0040-1709693

**Published:** 2020-05

**Authors:** Ismail Biyik, Mustafa Albayrak, Fatih Keskin

**Affiliations:** 1 Department of Obstetrics and Gynecology, School of Medicine, Kütahya Health Sciences University, Kütahya, Turkey; 2 Department of Obstetrics and Gynecology, Florence Nightingale Hospital, Istanbul, Turkey; 3 Private Balkan Hospital, Edirne, Turkey

**Keywords:** missed abortion, platelet to lymphocyte ratio, neutrophil to lymphocyte ratio, inflammation

## Abstract

**Objective** Missed abortion occurs in ∼ 15% of all clinical pregnancies. The pathogenesis is not clearly known. However, defective placentation resulting in maternal systemic inflammatory response is considered responsible for missed abortion. Platelet lymphocyte ratio (PLR) and neutrophil lymphocyte ratio (NLR) are increasingly cited parameters of inflammation in the literature. However, no study evaluated the PLR and NLR rates in missed abortions so far. The aim of the present study is to investigate whether complete blood count (CBC) inflammatory parameters such as NLR and PLR are increased in patients with missed abortion.

**Methods** Medical records of 40 pregnant women whose gestation ended in missed abortion at between 6 and14 weeks of gestation and of 40 healthy pregnant women were collected and compared retrospectively. The groups were compared regarding hemoglobin, hematocrit, platelet count (PLT), mean platelet volume (MPV), platelet distribution width (PDW), PLR and NLR.

**Results** Platelet distribution width, NLR and PLR values were higher in the missed abortion group compared with the healthy pregnant women group (rates are *p* = 0.043; *p* = 0.038; and *p* = 0.010, respectively). Hematocrit, MPV, and lymphocyte values were found to be lower in the missed abortion group compared with the healthy pregnant women group (*p* = 0.027, *p* = 0.044 and *p* = 0.025, respectively).

**Conclusion** The PDW, NLR and PLR values of the missed abortion group were reported high; and MPV values were reported low in the present study. These findings may help to speculate a defective placentation in the pathogenesis of missed abortion.

## Introduction

Missed abortion is defined as an embryonic part of the pregnancy left in the uterus after embryonic or fetal death. A total of 15% of all clinically diagnosed pregnancies result in abortion (miscarriage).^1^ Almost all of the 1^st^ trimester pregnancy losses occur following missed abortion.^2^ It is thought that chromosomal anomalies, immunological causes, uterine anomalies, endocrine causes and infections are responsible for the etiopathogenesis of missed abortion. However, the exact cause is not known. There are studies reporting that missed abortion may develop due to defective placentation with resultant maternal systemic inflammatory response.^3^ ^4^ ^5^ ^6^ ^7^ It is known that serum inflammatory cytokines interleukin 2 (IL-2), interleukin 12 (IL-12) and tumor necrosis factor α (TNF-α) increase in patients with missed abortion.^8^ ^9^

Platelet count (PLT), mean platelet volume (MPV), platelet distribution width (PDW), platelet lymphocyte ratio (PLR) and neutrophil lymphocyte ratio (NLR) are the parameters of complete blood count (CBC) for inflammation and/or ischemia. Neutrophil lymphocyte ratio is the ratio of absolute neutrophil count to the absolute lymphocyte count. It is regarded as a marker of the immune response of the body to offending agents. It is also regarded as a rapid and simple parameter indicative of systemic inflammation and stress.^10^ Platelet lymphocyte ratio is another parameter known to increase during thrombosis and inflammation.^11^ Mean platelet volume has been associated with thrombocyte volume, function, and activation and its increase is associated with the presence and prognosis of vascular disease, including peripheral, cerebrovascular, and coronary artery disease.^12^ ^13^ Along with MPV, platelet distribution width (PDW) is a marker of platelet activation.^14^ It is generally accepted that thrombocyte volume is determined during the production of thrombocytes from megakaryocytes.^15^ Larger thrombocytes are younger and less reactive.^16^

Some studies claim that pre-eclampsia and miscarriage (missed abortion) are similarly placenta-related diseases culminating in placental dysfunction, since both diseases have a common inflammatory component.^17^ Inflammatory markers such as NLR, PLR and MPV values are reported to increase in pre-eclampsia.^18^ Oylumlu et al^19^ claimed NLR as a prognostic marker to determine the systemic inflammatory response increase in pre-eclampsia. Therefore, we investigated whether the increase of these mediators are associated with missed abortion. However, to the best of our knowledge, no study evaluated NLR and PLR parameters in missed abortion so far.

The aim of the present study is to investigate whether CBC inflammatory parameters such as NLR and PLR are increased in patients with missed abortion.

## Methods

Ethical approval was obtained from the Ethics Committee of the SBU Bursa Yuksek Ihtisas Education and Research Hospital for this retrospective case-control study (2011-KAEK-25 2019/03–04). Permission was obtained from the patients for the study. The study was conducted in accordance with the Helsinki Declaration Principles. A total of 40 missed abortion patients and 40 matched healthy pregnant women at < 14 weeks of gestation admitted to the Gynecology and Obstetrics Clinic of the Bursa Karacabey State Hospital between January 2015 and December 2018 were included in the study. Demographic data such as age, previous gestations, parity, number of living children, height and weight were recorded.

A total of 80 women between the ages of 17 and 42 years old in between 6^0/7^ and 13^+6^ gestational week were included in the study. Missed abortion diagnosis was accepted as the loss of fetal heartbeat in a pregnant woman before the 14^th^ gestational week whose intrauterine fetal heart rate was detected previously. Additionally, women who had active vaginal bleeding with at least 6 weeks of pregnancy in which the heartbeat was undetectable were also accepted as missed abortion. Women with thyroid dysfunction, diabetes mellitus, hematologic disease, history of thrombosis, systemic lupus erythematosus, multiple pregnancy, smoking habits, active infection, malignancy, chronic inflammatory diseases (such as arthritis) or those on anti-inflammatory drugs or glucocorticoids were excluded from study. Those with detectable pathologies claimed to be involved in missed abortion pathophysiology, such as uterine anomalies, were excluded from the study. To eliminate the effect of obesity, only those with a body mass index (BMI) of < 25 kg / m^2^ were included in the study. Those with anembryonic pregnancy were excluded from the study.

Gestational age was determined based on the last menstrual period, which was confirmed by crown rump length (CRL) measurement on abdominal or transvaginal ultrasound. If the last menstrual period was uncertain or a discrepancy of > 1 week was detected between the last menstrual period or CRL measurement, the latter was accepted as true. Ultrasound imaging was performed by the same sonographer with the same device (Medison X8 Sonoace, Samsung Medison, Seoul, South Korea). All of the images were obtained by a 3.5 MHz convex transabdominal transducer or a 5 MHz vaginal probe was used when abdominal diagnosis could not be made reliably.

## Laboratory Parameters

Laboratory parameters such as hemoglobin, hematocrit, PLT, MPV, PDW, PLR, and NLR were recorded. Blood samples were drawn from the antecubital vein and collected in tubes containing ethylene diamine tetra acetic acid (K3EDTA). In the missed abortion group, blood was taken immediately after the diagnosis. The complete blood count was analyzed in an automatic full blood count machine (Cell-Dyn 3700, Abbott, Chicago, IL, USA).

## Statistical Analysis

Continuous variables were expressed as mean ± standard deviation (SD) or median (minimum: maximum) values. Categorical variables were expressed as n (%). The chi-squared test was used to compare categorical variables between groups. The independent *t*-test was used to compare continuous variables between groups. For statistical analysis, SPSS Statistics for Windows, Version 21.0 (IBM Corp., Armonk, NY, USA) was used. A *p*-value < 0.05 was considered statistically significant. A receiver operating characteristic (ROC) curve was constructed to determine the cutoff values of PDW and PLR for the diagnosis of missed abortion.

## Results

The two groups were similar in terms of maternal age, gestational age, body weight, BMI, gravidity, parity and number of living children (p > 0.005). The number of previous abortions was higher in the missed abortion group (*p* < 0.0001) (Table 1).

**Table 1:** Demographic parameters of groups

Variables	Missed abortion (*n* = 40)	Healthy pregnant (*n* = 40)	*p-value*
Age (years old)	29.27 ± 6.84	28.37 ± 5.13	0.508
Weight (kg)	66.54 ± 14.39	68.61 ± 15.89	0.543
Height (cm)	161.60 ± 6.67	162.72 ± 6.64	0.452
BMI (kg/m^2^)	25.47 ± 5.34	25.89 ± 5.71	0.732
Gravidity	2 (1–7)	2 (1–13)	0.557
Parity	1 (0–4)	1 (0–7)	0.440
History of abortion	1 (1–4)	0 (0–5)	< 0.0001
Living child	1 (0–4)	1 (0–5)	0.477
GA(days)	54.82 ± 11.54	54.12 ± 12.04	0.791

Abbreviations: BMI, Body mass index; GA, Gestational age.

Hemoglobin, platelet and neutrophil values were similar between the groups (p > 0.005). Platelet distribution width, NLR, and PLR values were higher in the missed abortion group (*p* = 0.043, *p* = 0.038 and *p* = 0.010, respectively). Hematocrit, MPV, and lymphocyte values of missed abortion patients were found to be lower compared with controls (*p* = 0.027, *p* = 0.044 and *p* = 0.025, respectively) (Table 2).

**Table 2:** Hematologic parameters of groups

Variables	Missed abortion (*n* = 40)	Healthy pregnant (*n* = 40)	*p-value*
Hemoglobin (g/dL)	12.62 ± 1.18	12.19 ± 0.96	0.075
Hematocrit (%)	38.11 ± 3.10	36.66 ± 2.64	0.027
Platelet (10^3^ / μl)	256.50 ± 76.27	253.63 ± 66.38	0.858
MPV (fL)	9.07 ± 1.48	9.72 ± 1.33	0.044
PDW (%)	16.65 ± 0.79	16.30 ± 0.73	0.043
Neutrophil (10^3^ / μl)	5.34 ± 1.89	5.30 ± 1.39	0.909
Lymphocyte (10^3^ / μl)	1.81 ± 0.57	2.13 ± 0.69	0.025
NLR	3.22 ± 1.69	2.60 ± 0.74	0.038
PLR	154.15 ± 66.85	123.72 ± 30.09	0.010

Abbreviations: MPV, mean platelet volume; NLR, neutrophil to lymphocyte ratio; PDW, platelet distribution width; PLR, platelet to lymphocyte ratio.

**Fig. 1:**
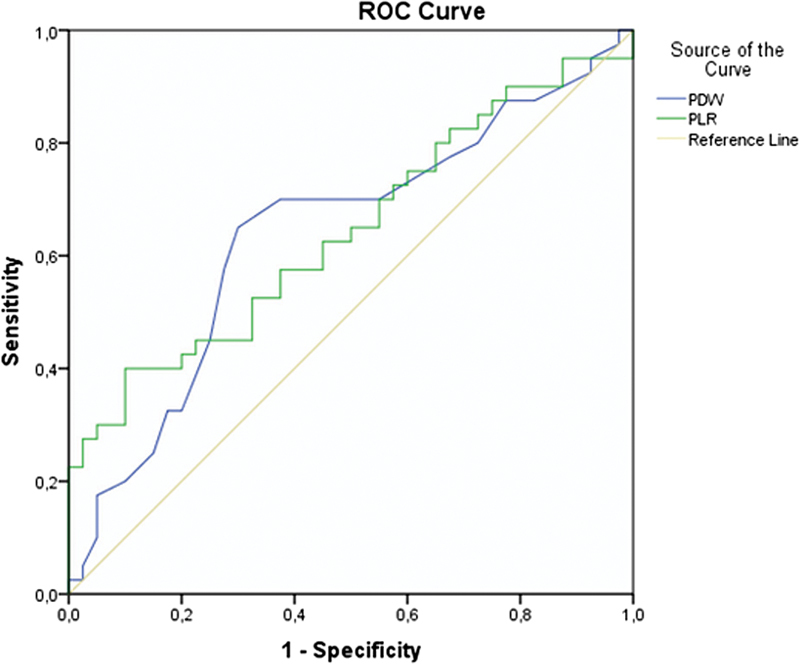
ROC curve analysis of complete blood count parameter for the determination of the diagnosis of missed abortion.

Receiver operating characteristic analysis was performed to determine diagnostic PDW and PLR values for missed abortion. A PDW value > 16.35 determined missed abortion with 70% sensitivity and 62.5% specificity. A PLR value > 123.14 determined miscarriage with 62.5% sensitivity and 55% specificity (Fig. 1).

## Discussion

The human fetus develops in a low-oxygen environment during the early stages of pregnancy. To maintain a low oxygen concentration, extravillous trophoblasts invade the uterine tissues and form a shell cell barrier into the ends of the uteroplacental arteries.^20^ This barrier protects the placenta during the early stages of pregnancy from the detrimental effects of free oxygen radicals formed as a result of early and excessive blood flow due to maternal circulation.^21^ Increased oxygen radicals cause necrosis and apoptosis in the placental villous tree of the trophoblast epithelium.^17^ Additionally, lipid peroxidation, which is also detrimental to invading trophoblastic tissue, also takes place in the placenta due to increased free oxygen radicals. Lipid peroxidation activates the cascade of biochemical events that causes leukocyte activation, adhesion and aggregation of platelets.^17^ Leukocyte activation causes an increase in inflammation parameters in blood and thrombosis resulting in ischemia of gestational tissues, which hinders the further development of the placenta. As a result of this defective placentation, maternal systemic inflammatory response increases as reflected in increased PLR and NLR.^22^

Studies on NLR, PLR and MPV have grown recently following the discovery of their immense values in the prediction and prognosis of many medical conditions. These parameters are potent markers of inflammation that underlies the basic pathologies of various diseases. The rapid availability of these parameters without additional costs to the patients may gradually replace the older markers of inflammation.

Jauniaux et al^17^ reported that the preeclampsia and loss of pregnancy (missed abortion) were placenta-related disease. In preeclampsia, inflammation and oxidative stress biomarkers increase in maternal blood.^23^ Among these increased inflammatory CBC markers, such as NLR, PLR, PDW and MPV, were shown to be elevated in pre-eclampsia.^18^ ^19^ ^24^ ^25^ ^26^ ^27^ The same model may also apply to missed abortion.

In the present study, PDW, NLR and PLR values of the missed abortion group were reported higher, and MPV values were reported lower compared with controls. We found only one study reporting the decrease in MPV in abortion. However, as far as we know, no study reported an increase in MPV in abortion compared with controls in the literature. Kosus et al^28^ found similar MPV values between healthy pregnant women and women with missed abortion. Bas et al^29^ found that MPV and PLR levels in the spontaneous abortion group were low; neutrophil, lymphocyte, NLR values were high in the same group. Eroglu et al^30^ reported no difference in MPV values between healthy pregnant women and women with missed abortion. However, to the best of our knowledge, there is no study to report PDW, NLR and PLR values in missed abortion compared with controls.

Our findings of high PDW, NLR and PLR values in missed abortion patients support the idea that claims a similarity between the ethiopathogenesis of missed abortion and pre-eclampsia. Increased NLR and PLR values in missed abortion may be the reflection of an increased maternal systemic response due to placentation disorder and trophoblast injury in the early stages of pregnancy, similar to pre-eclampsia. Likewise, we also think that high PDW values are a reflection of increased endothelial damage and risk of thrombosis as a result of defective placentation, a process similar to pre-eclampsia. It was previously shown that there is an increase in systemic inflammatory markers with increased TNF-α, interferon gamma and IL-2, IL-6, IL-10 and IL-12 levels in missed abortion.^8^ ^9^ ^31^ ^32^ The investigation of cytokinesis are expensive compared with the CBC parameters. However, CBC is almost routinely requested in the evaluation of missed abortion and does not add up additional cost if the CBC parameters may have any place. Experimental therapies directed at increased inflammatory response are investigated in pre-eclampsia,^33^ but no similar study was conducted for missed abortion. We think that our study may be another clue that increased inflammatory responses are operative in missed abortion. However, detailed and elaborative studies are needed to speculate that this inflammatory response may be an indirect sign of defective placentation in missed abortion as in the case of pre-eclampsia.

## Conclusion

In the present study, increased PDW, NLR and PLR and decreased MPV values in missed abortion were found compared with controls, which supports increased maternal systemic inflammatory response.
